# Evaluation of febrile seizures in children infected with SARS-CoV-2 Omicron variant in Yunnan, China: a multi-center, retrospective observational study

**DOI:** 10.3389/fped.2023.1223521

**Published:** 2023-11-13

**Authors:** Hai-feng Liu, Rui Lu, Jian Yang, Mei Xiang, Deng Ban, Jia-wu Yang, Zheng-hong Guo, Ting-yun Yuan, Hong-min Fu

**Affiliations:** ^1^ Department of Respiratory and Critical Care Medicine, Yunnan Key Laboratory of Children’s Major Disease Research, Kunming Children’s Hospital, Kunming Medical University, Kunming, China; ^2^ Department of Pediatrics, The People’s Hospital of Wenshan Zhuang & Miao Autonomous Prefecture, Wenshan, China; ^3^ Department of Pediatrics, The First People’s Hospital of Honghe Prefecture, Mengzi, China; ^4^ Department of Pediatrics, The First People’s Hospital of Zhaotong, Zhaotong, China

**Keywords:** Omicron variant, febrile seizures, coronavirus disease 2019, vaccination, children

## Abstract

**Background:**

The SARS-CoV-2 Omicron variant was reported to be linked to febrile seizures (FSs), but studies on FSs in children with Omicron infection remain relatively scarce, especially in the Chinese population. This study aimed to investigate the characteristics of children diagnosed with Omicron infection with FSs in Yunnan, China, and evaluate the potential association between FSs and Omicron infection.

**Methods:**

This study was conducted at four hospitals in Yunnan from December 8, 2022, to January 8, 2023, and consisted of 590 pediatric subjects. According to clinical characteristics, 85, 129 and 376 subjects were divided into the FS-only, Omicron-FS, and Omicron-only groups, respectively. Demographic, clinical and laboratory data were retrospectively collected for analysis.

**Results:**

The incidence of FSs in children with Omicron infection was 25.5% (129/505). Older age, stronger male predominance, as well as lower proportions of prior history and family history of seizures were observed in Omicron-FS and Omicron-only groups than in FS-only group, but there were no differences in these four above-mentioned events between these two Omicron-related groups. Compared to FS-only group, Omicron-FS group also had a shorter fever-to-seizure onset duration and more frequent seizures during a single course of fever. Moreover, higher levels of IL-6, TNF-α and ferritin as well as decreased counts of leukocytes and lymphocytes were confirmed in Omicron-FS group than in FS-only and Omicron-only groups. Regarding COVID-19 vaccination status, Omicron-FS group revealed a higher proportion of unvaccinated children and a lower proportion of three-dose vaccination than Omicron-only group. As for clinical outcomes, proportions of mechanical ventilation and intensive care unit admission observed in the two Omicron-related groups were notably higher than those in FS-only group. Meanwhile, Omicron-FS group showed the longest length of hospital stay, followed by Omicron-only group and FS-only group, in order. Finally, all patients but one who died of fulminant myocarditis had been successfully discharged.

**Conclusions:**

The incidence of FSs in children with Omicron infection was 25.5% in Yunnan. FSs might be a clinical sign deserving more attention in children with Omicron infection. Furthermore, COVID-19 vaccination is likely to provide effective protection against Omicron-related FSs in children.

## 1. Introduction

Over three years after the first case of SARS-CoV-2 infection was identified in December 2019 in Wuhan, China, 7.58 million infected individuals and 6.85 million deaths have been reported worldwide as of March 1, 2023 (1). As the coronavirus disease 2019 (COVID-19) pandemic continues, numerous genetically distinct lineages, including D614G, Beta/Gamma, Delta, and Omicron variants, have evolved. Among these variants, the Omicron variant, first identified in South Africa in early November 2021, has rapidly become the dominant strain circulating globally and accounts for approximately 99% of all current SARS-CoV-2 sequences in the GISAID database (2, 3). The Omicron variant, which is the most highly mutated strain with more than 50 mutations accumulated throughout its genome, might make conventional vaccines, drugs, and coronavirus measures less effective and pose substantial challenges to public health systems worldwide. Compared to other SARS-CoV-2 variants, the prevalence of COVID-19 caused by the Omicron variant is higher in all age groups, especially in children. According to some recent publications, the Omicron variant is driving the current surge of pediatric COVID-19 cases in various countries around the world, and the peak rate of COVID-19–associated hospitalization among children during the Omicron-predominant period was estimated to be four times as high as the peak rate during the Delta period (4, 5). A national commercial laboratory seroprevalence study from the United States indicated that as of February 2022, approximately 75% of children and adolescents were confirmed to have previous SARS-CoV-2 infection, with approximately 33% becoming newly seropositive since December 2021, and the Omicron variant contributed more than 92% of these new cases (6). Fortunately, pediatric patients admitted to the hospital with the Omicron variant seemed to show significantly less severe outcomes than patients with other SARS-CoV-2 variant infections, as they required less medical support and shorter hospital stays (7). Although most literature about the clinical features of Omicron infection in the pediatric population has reported fever (62.8%), cough (59.3%) and nasal discharge (53.5%), several neurological manifestations, ranging from common to rare, have also been reported (8, 9). Regarding neurologic events in pediatric COVID-19 patients, previous research has placed more emphasis on cognitive deficits, ischemic stroke, psychotic disorders, and headaches. In contrast, febrile seizures (FSs) were not viewed as a common condition of COVID-19 before the Omicron surge, with a low incidence rate of only 0.5%–6% (10, 11).

In children, FSs are a common neurologic event with an incidence of 2%–5%, characterized by a fever that occurs in a child <5 years without evidence of a central nervous system (CNS) infection (12). During the Omicron wave, FSs seemed to be more frequent in children with COVID-19. According to Iio et al. (13), FSs were observed in five pediatric Omicron patients who were admitted to the Tokyo Metropolitan Children's Medical Center during the initial Omicron wave, which was the first report of children with Omicron infection who developed FSs in Asia. Subsequently, a single-center retrospective observational study from Japan indicated that 14.2% of pediatric COVID-19 cases during the Omicron era in Tokyo had FSs (14). Virus-induced cytokine storms and action on the CNS have been suggested to generate neuronal hyperexcitability, thus causing FSs (15). Specifically, neurotropic and neuroinvasive capabilities of SARS-CoV-2 have been described in several studies due to the presence of angiotensin-converting enzyme 2 (ACE2) in neurons, astrocytes, and oligodendrocytes (16, 17). SARS-CoV-2 can utilize the spike protein to recognize and bind to ACE2 in neural cells, which mediates viral invasion of the CNS through transsynaptic transfer, the transneuronal olfactory bulb pathway, hematogenous routes and rupture of the blood‒brain barrier (BBB), thus resulting in excessive immune reactions and inflammation and eventually leading to the development of a series of neurological problems, including FSs (18–20). Owing to the numerous mutations in the spike protein, the Omicron variant was endowed with a stronger affinity for ACE2 and might therefore cause a higher potential risk of FSs.

However, only a limited number of studies reporting FSs in pediatric Omicron patients are available (13, 21, 22), and there is still a paucity of research regarding the specific incidence and characteristics of Omicron-related FSs in children, especially in the Chinese population. Hence, we retrospectively analyzed the demographic, clinical and laboratory data of a Chinese group of pediatric COVID-19 patients with FSs during the current Omicron wave in Yunnan and compared them to children diagnosed with traditional FSs without SARS-CoV-2 infection and children diagnosed with COVID-19 without seizures. This study aimed to identify the incidence and characteristics of FSs in children with Omicron infection, provide a scientific basis for investigating the potential association between FSs and Omicron infection, and inform clinical practice.

## 2. Materials and methods

### 2.1. Study design and subjects

This study was a retrospective, multi-center study conducted at four tertiary A hospitals in Yunnan, including Kunming Children's Hospital, the People's Hospital of Wenshan Zhuang & Miao Autonomous Prefecture, the First People's Hospital of Honghe Prefecture, and the First People's Hospital of Zhaotong. The study population comprised of three pediatric groups of subjects aged 0–14 years who visited the above four hospitals, from December 8, 2022 to January 8, 2023, when Omicron was the predominant strain and accounted for over 99% of all SARS-CoV-2 variants circulating in Yunnan (https://www.yncdc.cn/newsView.aspx?id=133382). The first was the FS-only group, which enrolled children diagnosed with FSs without SARS-CoV-2 infection. The second was the Omicron-FS group, which comprised pediatric COVID-19 patients with FSs. The third was the Omicron-only group, which consisted of children diagnosed with COVID-19 without FSs. All diagnoses of COVID-19 were confirmed by reverse transcription-polymerase chain reaction (RT‒PCR) for SARS-CoV-2 using nasopharyngeal swab samples, and all COVID-19 patients in this study were presumed to be infected with Omicron due to the overwhelming dominance of the Omicron variant during the current COVID-19 pandemic in Yunnan. FSs were defined as seizures accompanied by fever (temperature ≥ 38°C) without intracranial infection or defined cause according to the guidelines from the American Academy of Pediatrics (AAP) (12). Meanwhile, the exclusion criteria were as follows: (1) patients with intracranial space-occupying lesions; (2) patients who had already been diagnosed with epilepsy; (3) COVID-19 cases coinfected with other pathogens; or (4) patients with incomplete or unavailable data. Ethical approval was obtained from the Ethics Committees of the four hospitals. The study protocol was conducted in accordance with applicable local laws and regulatory requirements as well as the principles of the Declaration of Helsinki. Informed consent was waived as part of a public health outbreak investigation.

### 2.2. Data collection

Detailed data including demographic information, prior history and family history of seizures, clinical characteristics, laboratory indicators [such as complete blood count, C-reactive protein (CRP), inflammatory cytokines, serum biochemical analysis, etc.], COVID-19 vaccination status, and clinical outcomes [including mechanical ventilation, intensive care unit (ICU) admission, length of hospital stay (LOS), and discharge with cure or improvement] were retrospectively collected from electronic medical records.

### 2.3. Statistical analysis

Statistical processing and graphing were conducted using SPSS 26.0 (IBM Corp, Armonk, NY, USA) and GraphPad Prism 7.0 (GraphPad Software, San Diego, CA, USA). Nonnormally distributed quantitative variables are presented as the median and interquartile range [M (Q1-Q3)]. Comparisons between the two groups were examined by the Mann‒Whitney test, and comparisons among multiple groups were performed by the Kruskal-Wallis test. Additionally, qualitative data are expressed as percentages and were compared utilizing the chi-square test or Fisher's exact test. All multiple comparisons were adjusted using the Bonferroni correction. For all analyses, a *P* value of <0.05 was considered to be statistically significant.

## 3. Results

### 3.1. Study population and demographic data

**Figure 1:**
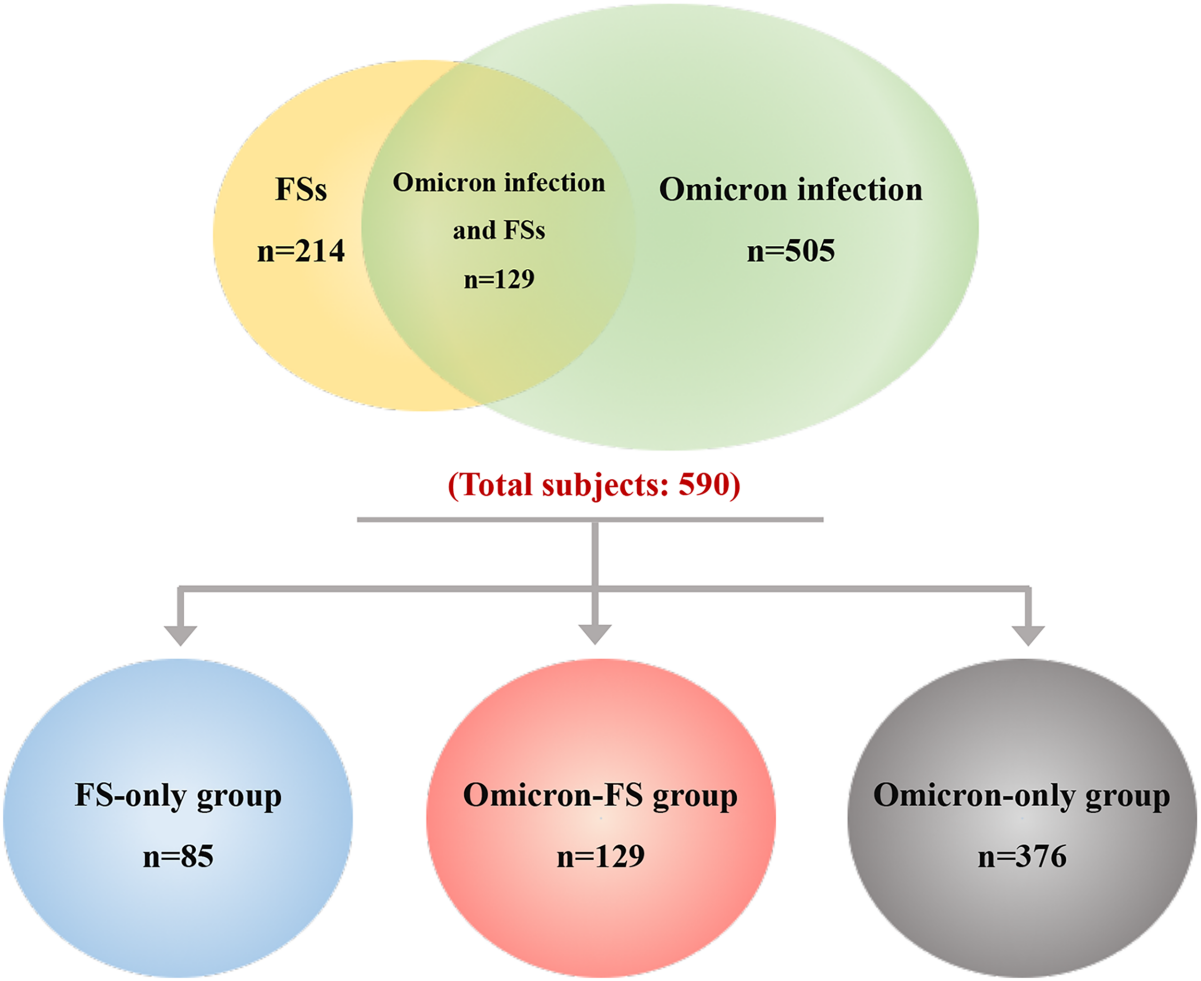
Overall distribution and grouping of subjects. A total of 590 pediatric patients were included, and of these, 214 were found to have FSs (marked in yellow and containing overlapping region) and 505 had Omicron infection (marked in green and containing overlapping region). The overlapped region indicated that 129 subjects were diagnosed with Omicron infection and FSs among these patients. According to the design of this study and classification of diseases, these 590 subjects were divided into three groups, including 85 in the FS-only group (marked in blue), 129 in the Omicron-FS group (marked in red), and another 376 in the Omicron-only group (marked in gray).

During the entire study period, a total of 590 pediatric subjects evaluated for eligibility were included. Of these, 214 subjects were found to have FSs and 505 subjects had Omicron infection. On the other hand, 129 subjects were diagnosed with Omicron infection and FSs among these patients. That is, the incidence of FSs in children with Omicron infection was approximately 25.5% (129/505). According to the design of this study and classification of diseases, these 590 subjects were assigned into three groups, including 85 in the FS-only group, 129 in the Omicron-FS group, and another 376 in the Omicron-only group (Figure 1).

As presented in Table 1, significant differences in age and sex were observed among these three groups (both *P *< 0.001). Specifically, in the FS-only group, the median age was 2.3 (Q1–Q3, 1.6–3.4) years, and 45 patients (52.9%) were male. In the Omicron-FS group, the median age was 5.9 (Q1–Q3, 4.1–6.7) years, and 94 patients (72.9%) were male. In the Omicron-only group, the median age was 5.7 (Q1–Q3, 4.0–6.7) and 264 patients (70.2%) were male. Compared to the FS-only group, children from the two Omicron groups were older (5.9 years vs. 2.3 years, *P *< 0.05; 5.7 years vs. 2.3 years, *P *< 0.05) and showed apparent male predominance [72.9% (94/129) vs. 52.9% (45/85), *P *< 0.05; 70.2% (264/376) vs. 52.9% (45/845), *P *< 0.05]. Nevertheless, the distributions of age and sex were equal between the two Omicron groups (both *P *> 0.05). Additionally, 16 (18.8%), 25 (19.4%) and 76 (20.2%) subjects were confirmed to be Chinese minorities in the FS-only, Omicron-FSs and Omicron-only groups, respectively, and no ethnic difference was exhibited among these three groups (*P *= 0.949).

**Table 1:** Characteristics of all subjects.

	FS-only group	Omicron-FS group	Omicron-only group	*χ*2/Z/H	*P* value
	*n *= 85	*n *= 129	*n *= 376		
Age, y, median (Q1–Q3)	2.3 (1.6–3.4)	5.9 (4.1–6.7)*	5.7 (4.0–6.7)*	116.683	<0.001
Sex, male, *n* (%)	45 (52.9)	94 (72.9)*	264 (70.2)*	11.141	<0.001
Chinese minorities, *n* (%)	16 (18.8)	25 (19.4)	76 (20.2)	0.105	0.949
Prior history of seizures, *n* (%)	51 (60.0)	24 (18.6)*	73 (19.4)*	64.455	<0.001
Family history of seizures, *n* (%)	52 (61.2)	16 (12.4)*	44 (11.7)*	114.985	<0.001
Fever duration before seizure onset, h, median (Q1–Q3)	8.0 (8.0–9.0)	5.0 (5.0–6.0)	NA	10.978	<0.001
Fever peak, °C, median (Q1–Q3)	39.1 (38.8–39.5)	39.0 (38.8–39.5)	NA	0.495	0.621
Seizure frequency during single course of fever, time, median (Q1–Q3)	1.0 (1.0–1.0)	2.0 (1.0–3.0)	NA	6.629	<0.001
Semiology of seizures					
Seizure patterns, *n* (%)					
Generalized tonic-clonic	81 (95.3)	122 (94.6)	NA	0.055	0.815
Tonic	0	0	NA	NA	NA
Clonic	0	0	NA	NA	NA
Atonic	0	0	NA	NA	NA
Focal-onset	4 (4.7)	7 (5.4)	NA	0.055	0.815
Duration, min, median (Q1–Q3)	3.0 (1.0–6.0)	3.0 (2.0–5.0)	NA	0.954	0.340

FSs, febrile seizures; NA, not applicable.

* *P *< 0.05, compared to FS-only group.

### 3.2. Clinical features

As shown in Table 1, of the 85 patients in the FS-only group, 51 (60.0%) and 52 (61.2%) had a prior history and family history of seizures, respectively. Compared to FS-only group, both Omicron-FS group and Omicron-only group showed significantly lower proportions of prior history and family history of seizures [18.6% (24/129) and 12.4% (16/129) in Omicron-FS group, respectively; 19.4% (73/376) and 11.7% (44/376) in Omicron-only group, respectively], despite no marked differences in the above two events between the two Omicron-related groups (both *P *> 0.05). A comparison of seizure semiology and clinical information between the FS-only group and the Omicron-FS group was also conducted. Children from the FS-only group had a longer fever-to-seizure onset duration (8.0 vs. 5.0 h, *P *< 0.001) and a lower frequency of seizure episodes during the single course of fever (1.0 vs. 2.0 times, *P *< 0.001) than those in the Omicron-FS group. Additionally, there were no significant differences between the two groups regarding the fever peak, seizure patterns and duration of seizures (all *P *> 0.05).

### 3.3. Laboratory characteristics

**Figure 2:**
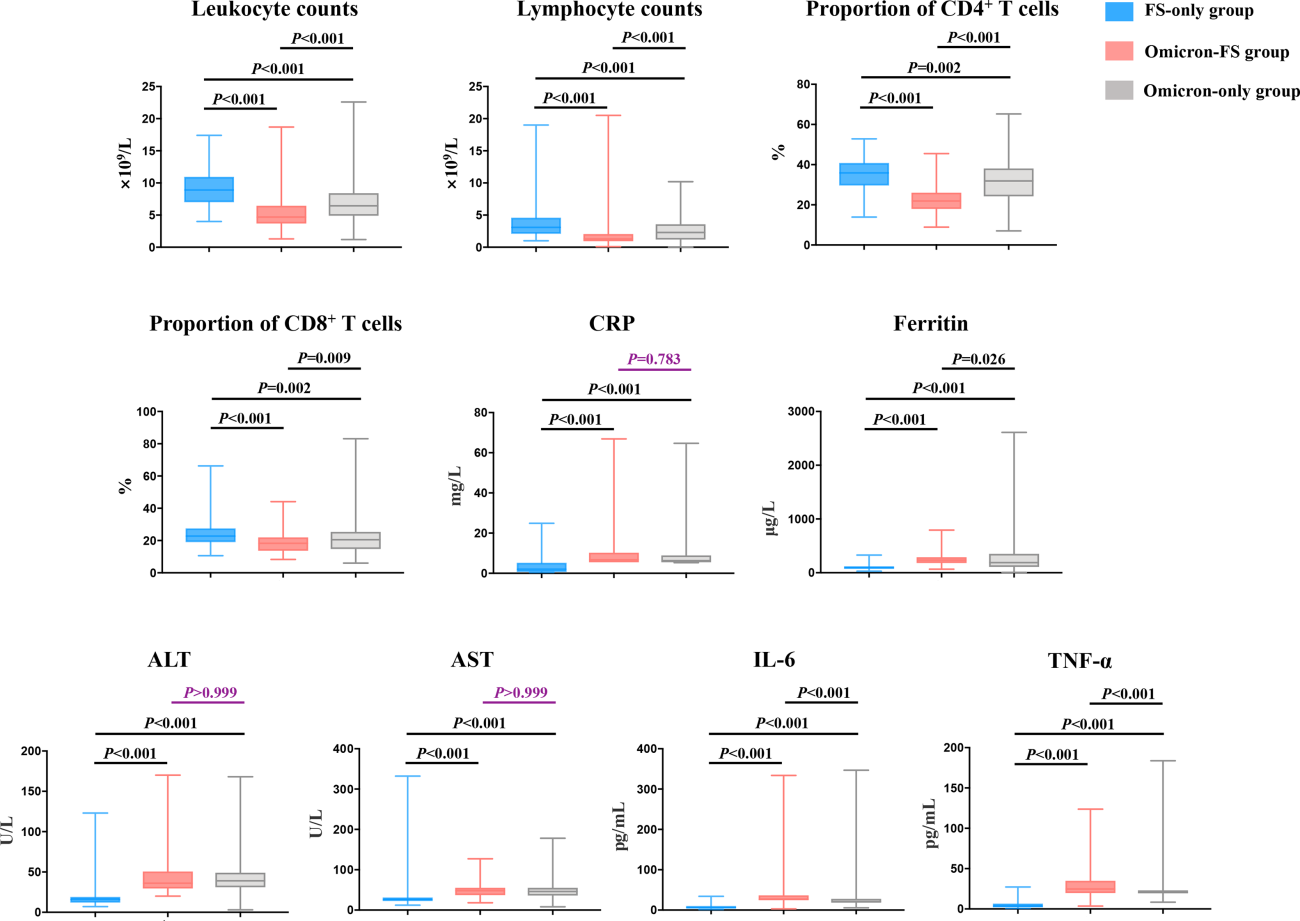
Visualization and comparison of laboratory characteristics among the three groups. Except for CRP (*P *= 0.783), ALT (*P *> 0.999) and AST (*P *> 0.999) between Omicron-FS and Omicron-only groups, all pairwise comparisons of leukocyte counts, lymphocyte counts, the proportion of CD4^+^ and CD8^+^ T cells, CRP, ferritin, ALT, AST, IL-6 and TNF-α among the three groups were statistically significant. *P* values > 0.05 were marked in purple.

Routine laboratory parameters of all subjects were evaluated, and significant differences in leukocyte counts, lymphocyte counts, the proportion of CD4^+^ and CD8^+^ T cells, C-reactive protein (CRP), ferritin, alanine aminotransferase (ALT), aspartate aminotransferase (AST), IL-6, and TNF-α were observed between the three groups (all *P *< 0.001, Table 2). The results of pairwise comparisons among the multiple groups were visualized by GraphPad Prism software to show differences between the various groups more clearly (Figure 2). Compared to the FS-only group, both the Omicron-FS and Omicron-only groups indicated prominent decreases in leukocyte counts, lymphocyte counts and the proportion of CD4^+^ T and CD8^+^ T cells and increases in the serum levels of CRP, ALT, AST, IL-6, and TNF-α (Figure 2). Intriguingly, the corresponding degree of elevation or decline in the above indicators, except for CRP, ALT, and AST, was more significant in the Omicron-FS group than in the Omicron-only group. In other words, no marked difference in CRP (6.7 vs. 6.3 mg/L, *P *= 0.783), ALT (36.0 vs. 39.0 U/L, *P *> 0.999) and AST (48.0 vs. 46.0 U/L, *P *> 0.999) were existed between the Omicron-FS and Omicron-only groups (Figure 2).

**Table 2:** The main laboratory indicators of all subjects.

	FS-only group	Omicron-FS group	Omicron-only group	*H*	*P* value
	*n* = 85	*n* = 129	*n* = 376		
Leukocyte counts, × 10^9^/L, median (Q1–Q3)	8.9 (7.0–11.0)	4.7 (3.7–6.5)	6.5 (4.9–8.4)	104.717	<0.001
Lymphocyte counts, × 10^9^/L, median (Q1–Q3)	3.1 (2.1–4.6)	1.3 (1.0–2.1)	2.3 (1.2–3.6)	77.130	<0.001
Proportion of CD4^+^ T cells, %, median (Q1–Q3)	35.9 (29.7–40.8)	21.9 (18.0–26.1)	31.9 (24.2–38.1)	105.232	<0.001
Proportion of CD8^+^ T cells, %, median (Q1–Q3)	22.8 (19.0–27.5)	18.3 (13.7–22.0)	20.5 (14.8–25.4)	25.749	<0.001
CRP, mg/L, median (Q1–Q3)	2.0 (0.7–5.3)	6.7 (5.5–10.2)	6.3 (5.5–8.9)	106.074	<0.001
Ferritin, μg/L, median (Q1–Q3)	98.2 (70.2–119.5)	233.3 (181.3–288.6)	187.5 (108.6–350.3)	88.980	<0.001
ALT, U/L, median (Q1–Q3)	16.0 (12.0–19.0)	36.0 (29.5–50.5)	39.0 (31.0–49.0)	163.351	<0.001
AST, U/L, median (Q1–Q3)	26.0 (22.5–31.0)	48.0 (37.0–55.0)	46.0 (36.0–55.0)	130.459	<0.001
IL-6, pg/ml, median (Q1–Q3)	6.3 (4.3–10.3)	27.3 (24.6–36.6)	20.9 (18.6–27.8)	220.059	<0.001
TNF-α, pg/ml, median (Q1–Q3)	3.5 (2.1–6.5)	24.7 (19.7–35.0)	20.5 (19.4–23.1)	204.336	<0.001

ALT, alanine aminotransferase; AST, aspartate aminotransferase; CRP, C-reactive protein.

### 3.4. COVID-19 vaccination status

There were notable differences between patients who received zero and three doses of vaccination between the Omicron-FSs and Omicron-only groups (Table 3). Among the 129 children from the Omicron-FS group, 90 patients (69.8%) were unvaccinated, and 5 patients (3.9%) completed three-dose vaccination, which were significantly higher and lower than those in the Omicron-only group, respectively [69.8% (90/129) vs. 37.8% (142/376), *P *< 0.001; 3.9% (5/129) vs. 37.0% (139/376), *P *< 0.001]. Single- and two-dose vaccinations did not differ significantly between the two groups. On the other hand, differences were also observed between the four groups with different vaccination statuses ranging from zero to three doses of vaccines. Of concern was the finding that the proportion of the zero-dose group was much higher than that of the three-dose group in the Omicron-FS group [69.8% (90/129) vs. 3.9% (5/129), *P *< 0.05], while no difference was identified among the zero-dose and three-dose groups in the Omicron-only group (*P *> 0.05).

**Table 3:** COVID-19 vaccination status in children with omicron infection.

	Omicron-FS group	Omicron-only group	*χ^2^*	*P* value
	*n* = 129	*n* = 376		
Zero-dose vaccination, *n* (%)	90 (69.8)	142 (37.8)	39.606	<0.001
One-dose vaccination, *n* (%)	7 (5.4)	13 (3.5)	0.979	0.332
Two-dose vaccination, *n* (%)	27 (20.9)	82 (21.8)	0.044	0.834
Three-dose vaccination, *n* (%)	5 (3.9)*,***	139 (37.0)**,***	51.600	<0.001
*χ^2^*	196.083	156.511		
*P* value	<0.001	<0.001		

* *P *< 0.05, compared to zero-dose vaccination group.

** *P *< 0.05, compared to one-dose vaccination group.

*** *P *< 0.05, compared to two-dose vaccination group.

### 3.5. Clinical outcomes

The clinical outcomes of all patients were summarized in Table 4. Compared to the FS-only group [1.2% (1/85) and 2.4% (2/85), respectively], there were significantly more patients requiring mechanical ventilation and ICU admission in the Omicron-FS group [10.9% (14/129) and 14.7% (19/129), respectively] and Omicron-only group [9.3% (35/376) and 11.2% (42/376), respectively] while no marked differences were observed between these two Omicron-related groups (*P *= 0.078). In terms of LOS, significant differences existed among these three groups. The Omicron-FS group showed the longest LOS with a median of 7.0 (Q1–Q3, 6.0–8.0) days, followed by 5.0 (Q1–Q3, 4.0–6.0) days in the Omicron-only group and 3.0 (Q1–Q3, 2.0–3.0) days in the FS-only group, in order. Finally, all patients in these three groups but one (from the Omicron-only group) who died of fulminant myocarditis were cured or improved [100% (85/85) in the FS-only group, 100% (129/129) in the Omicron-FS group, and 99.7% (375/376) in the Omicron-only group, respectively].

**Table 4:** Clinical outcomes of all subjects.

	FS-only group	Omicron-FS group	Omicron-only group	*χ^2^/H*	*P* value
	*n* = 85	*n* = 129	*n* = 376		
Mechanical ventilation, *n* (%)	1 (1.2)	14 (10.9)*	35 (9.3)*	7.115	0.029
ICU admission, *n* (%)	2 (2.4)	19 (14.7)*	42 (11.2)*	8.491	0.014
LOS, d, median (Q1–Q3)	3.0 (2.0–3.0)	7.0 (6.0–8.0)*,**	5.0 (4.0–6.0)*,**	224.813	<0.001
Discharge with cure or improvement, *n* (%)	85 (100.0)	129 (100.0)	375 (99.7)	1.137	1.000

ICU, intensive care unit; LOS, length of hospital stay.

* *P *< 0.05, compared to FS-Only group.

** *P *< 0.05, comparison between Omicron-FS and Omicron-only groups.

## 4. Discussion

After the emergence of the SARS-CoV-2 Omicron variant designated as a variant of concern (VOC) by the World Health Organization (WHO), it spread rapidly across the globe, and an increased number of COVID-19 infections in the pediatric population was observed during the Omicron wave, including in recent months in China due to the new guidelines issued by the Chinese government in December 7, 2022 that aimed to lift the strict “zero-COVID-19” policy, despite increasing economic and quality-of-life benefits from the new guidelines (23, 24). FSs seemed to emerge more frequently in children infected with the Omicron variant than in those infected with the original SARS-CoV-2 strain and previous variants. However, reports on FSs in pediatric Omicron patients are relatively scarce, particularly in the Chinese population. Therefore, we designed three groups, thus investigating the frequency and characteristics of FSs in children infected with Omicron during the current Omicron surge in Yunnan, China, and evaluating the possible links between FSs and Omicron infection in children. To the best of our knowledge, this is the first study regarding the characteristics of FSs associated with Omicron infection in Chinese children.

Demographic characteristics of the three populations were analyzed in the present study. First, our study showed an apparent difference between the two Omicron-related groups (Omicron-FS and Omicron-only groups) and the FS-only group with regard to age, but no age difference was revealed between the Omicron-FS and Omicron-only groups. This finding was consistent with previous research that pointed out the younger age of pediatric FS patients compared to that of children with Omicron infection. FSs mostly develop in children under 5 years of age and have a peak incidence within 24 months of life (25), while approximately one-third of the children infected with Omicron were older than 5 years (14). According to Yu et al. (26), the mean age of FSs was 28.3 months, which was significantly lower than that of pediatric Omicron cases (5.7 years) reported from China by Li et al. (27), whose finding was extremely close to ours. Second, a significant male predominance was noted in both the Omicron-FS and Omicron-only groups, while the distribution of sex was equal in the FS group. Male predominance has been widely reported as a general characteristic of adult COVID-19 patients and attributed to male social behavior and interaction, but these factors were not present in children (28). Similar to our findings, two recent reports showed that the proportion of males in pediatric Omicron cases was 54.8% in Shanghai, China and 70.7% in Korea (29, 30). Although not completely elucidated, sex hormones, including estrogen and testosterone, could be involved in the regulation of virus-driven T-cell differentiation, thus influencing antiviral immune responses, which are considered possible explanations for this gender disparity (31, 32). In addition, discrepancy in the innate immunity represented by the mononuclear phagocyte system was also suggested as a potential factor of gender difference in this disease (33). In addition, fewer than one-fifth of the patients had a prior history (18.6%) or family history (12.4%) of seizures in our Omicron-FS group, and these proportions were prominently lower than those (60.0% and 61.2%, respectively) in the FS-only group (18.6% vs. 60.0%, *P *< 0.001; 12.4% vs. 61.2%, *P *< 0.001, respectively). The significant difference in the rates of prior history and family history of seizures between the two groups might reflect the underlying role of Omicron infection in the occurrence of FSs.

More remarkably, in this study, the incidence of FSs (25.5%) in children with Omicron infection was higher than that in previous reports, and a greater number of episodes of seizures during a single course of fever (2.0 vs. 1.0, *P *< 0.001) as well as a shorter fever-to-seizure onset duration (5.0 h vs. 8.0 h, *P *< 0.001) were confirmed in the Omicron-FS group than in the FS-only group. Despite the limited direct evidence, FSs are currently regarded as one of the main presentations of acute Omicron infection due to their increased frequency reported in some areas (30). According to a multicenter observational study of the pediatric population with COVID-19 in South Africa, approximately 20% of children with COVID-19 during the Omicron wave were found to develop FSs, and this was slightly lower than that of our results (23). Genetic predisposition and environmental factors probably accounted for the observed differences between our study and the study conducted in South Africa because Asians have been reported to have a higher risk of seizures than other populations (34). The present study was performed in Yunnan, one of the main gathering places of Chinese ethnic minorities in China. In our three groups, nearly 20% of the patients belonged to ethnic minorities. More complex genetic factors related to seizures might exist due to the special customs of some ethnic minorities, such as consanguineous marriage.

In addition, the incidence of FSs in children with Omicron infection in this study was significantly higher than that in the pre-Omicron period. A large retrospective database study in the United States showed that only 0.5% of pediatric COVID-19 subjects were diagnosed with FSs before the Omicron wave (10). This rate in Japan and the United Kingdom was only 1.7% and 5.5%, respectively, during the early COVID-19 period when the Omicron variant had not emerged (14, 35). There was at least an approximately 4.6-fold difference between our results and the data from previous research conducted in the pre-Omicron era, suggesting that FSs might be gradually becoming a common manifestation associated with Omicron infection in the pediatric population. The discrepancy could also be explained by the genomic epidemiology of SARS-CoV-2. According to the data from the GISAID database, the original mutant strain of Omicron and previous SARS-CoV-2 variants diverged early in the COVID-19 pandemic, revealing that the Omicron variant was substantially distinct from other strains (36).

As we have mentioned earlier in this paper, a potential reason for the increased incidence of FSs in children with Omicron infection could be that numerous mutations in the spike protein of Omicron strengthened the binding affinity of the virus spike protein for ACE2 of neural cells, facilitating viral invasion of the CNS (16, 37). In addition to the direct CNS invasion of the virus, CNS involvement in COVID-19 patients has also been linked to the induction of excessive cytokine secretion, which has also been perceived as a clue to the potential mechanism relevant to FSs in Omicron infection (38) and partially illustrated the higher levels of IL-6 and TNF-α revealed in the Omicron-FS group than in the Omicron-only and FS-only groups in our study. As one of the most critical factors in the infection process of SARS-CoV-2, the host immune system is deeply implicated in the development of COVID-19 (39). During viral invasion, cytokines are activated pathologically due to excessive immune responses, thus generating the so-called cytokine storm, which can damage the BBB, increase the entry of inflammatory mediators and aggravate inflammatory injury of the CNS. Consistent with our observation, Korobova et al. (40) also described a rise in serum levels of inflammatory cytokines (including IL6 and TNF-α) in COVID-19 patients.

Meanwhile, excessive release of the above inflammatory mediators and related mechanisms could help to explain another phenomenon that compared to the Omicron-only and FS-only groups, the counts of leukocytes and lymphocytes, including CD4^+^ and CD8^+^ T cells, were markedly reduced while the serum ferritin level was significantly increased in the Omicron-FS group. First, a large number of clinical studies have indicated that high levels of cytokines and changes in circulating leukocyte subsets, particularly CD4^+^ and CD8^+^ T cells, are closely related to the progression of COVID-19 (41, 42). Indeed, Wang et al. (43) reported that elevated cytokine secretion occurred prior to the decline in CD4^+^ and CD8^+^ T cells. SARS-CoV-2 infection could induce the excessive release of cytokines due to immune dysregulation, thereby inhibiting the proliferation and activation of T lymphocytes through a negative feedback mechanism and resulting in delayed viral clearance (42). Second, as already mentioned, a significant difference in serum ferritin level was also identified between the three groups, among which it was highest in the Omicron-FS group. As a consequence of uncontrolled inflammatory responses, ferritin is secreted by hepatocytes and macrophages under the trigger of excessive proinflammatory factors such as IL-6 and TNF-α (44). It has been previously shown that serum ferritin is sensitive and specific for the early evaluation of COVID-19 severity (45). Based on the above obvious differences in several laboratory parameters among the Omicron-FS group and the other two groups, we tentatively speculated that high levels of proinflammatory cytokines (IL-6 and TNF-α), their associated impairment of immune cells and elevated serum ferritin levels might be closely linked to FSs in children with Omicron infection. Moreover, the serum levels of ALT, AST, and CRP in both the Omicron-FS and Omicron-only groups were moderately increased compared with those in the FS-only group, but we did not deem these three elevated parameters to be the potential drivers for FSs associated with Omicron infection because no difference in these three parameters was found between the Omicron-FS and Omicron-only groups.

Several studies have speculated that seizures are only indirectly triggered by SARS-CoV-2 infection rather than by direct viral invasion of the CNS (14). However, SARS-CoV-2 RNA was recently identified to be able to be widely distributed in the brain tissues of COVID-19 patients by Stein et al. (16). Hence, combined with the above statistics and analyses as well as the report of Stein et al., it was reasonable to speculate that FSs in pediatric Omicron cases might be driven by direct viral invasion in combination with inflammatory impairment induced by the penetration of overproduced proinflammatory mediators into the CNS rather than only indirectly triggered by SARS-CoV-2 (14). In addition, we also suggested that FSs were likely to be one of the clinical signs for Omicron infection in children due to its higher incidence during the current Omicron wave than other SARS-CoV-2 strains and its different characteristics compared with traditional FSs. Perhaps the atypical age, male predominance, relatively lower rates of prior history and family history of seizures, more frequent episodes of seizures during a single course of fever, a shorter fever-to-seizure onset duration and abnormal laboratory indicators in the Omicron-FS group might reflect the different mechanisms underlying FSs in children with Omicron infection, as opposed to those underlying traditional FSs.

COVID-19 vaccination status was also analyzed to investigate whether vaccines would confer protection against Omicron-related FSs. Obviously, the vaccination status in the Omicron-only group was better than that in the Omicron-FS group. Briefly, the Omicron-only group had a significantly higher proportion of three-dose vaccinations (37.0% vs. 3.9%, *P *< 0.001) and a lower proportion of unvaccinated cases (37.8% vs. 69.8%, *P *< 0.001) than the Omicron-FS group. Meanwhile, unvaccinated patients (69.8%) were significantly predominant among the four groups in the Omicron-FS group, but this was not observed in the Omicron-only group, in which 37.8% of patients were unvaccinated. On the other hand, there were no significant differences between the Omicron-FS group and Omicron-only group regarding prior history and family history of seizures, which are closely related to FSs. Thus, we speculated that COVID-19 vaccination, especially a booster dose, may reduce the occurrence of FSs in children with Omicron infection. Echoing our speculation, Tso et al. (46) recently reported that no or incomplete COVID-19 vaccination may be associated with the occurrence of CNS complications (including FSs) in children infected with the Omicron variant.

Finally, in the terms of outcomes, almost all patients had recovered and been successfully discharged from hospitals. However, although no significant differences in discharge were observed among these three groups, we found notably higher proportions of mechanical ventilation and ICU admission as well as longer LOS in the two Omicron-related groups compared to the FS-only group. Furthermore, LOS in the Omicron-FS group was also markedly longer than that in the Omicron-only group, despite no differences in remaining three outcome indicators between these two groups. These data suggested that the final clinical outcomes of patients in these three groups were generally favorable, but children with Omicron-related FSs seemed to have a more severe clinical course than those with traditional FSs or common type of Omicron infection.

Indeed, our study had several limitations. As this was a retrospective study, only associations rather than causal relationships could be inferred from our findings. Complete sequence information of SARS-CoV-2 strains in patients was unavailable. Meanwhile, due to the latency of Omicron infection, a few patients with a false-negative result of RT-PCR for SRAS-CoV-2 might be mistakenly assigned to the FS-only group. Whereas, during the study period, which was the early stage after lifting COVID-19 restrictions in China, rapid SARS-CoV-2 antigen self-testing kits were widely and frequently self-applied by the general public at home or in hospitals regardless of whether they have COVID-19-related symptoms. This measure reduced potential false-negative cases to some extent, guaranteeing the accuracy of grouping. Another limitation was the potential recall bias because partial semiology of the seizures and the duration of fever-to-seizure onset were provided by the guardians. Additionally, neutralizing activities of the antibodies induced by COVID-19 vaccines in patients were also unavailable, thus limiting more in-depth investigation about the clear protective efficacy of vaccines for seizures in children infected with the Omicron variant. Finally, when discussing whether COVID-19 vaccination can provide protection against FSs, the relationship between vaccination status and prior history of seizures also needs to be considered because of some vaccination contraindication, such as uncontrolled seizures, while data on this aspect were limited in our study.

## 5. Conclusions

FSs were more common (25.5%) in pediatric COVID-19 patients with Omicron infection than in children infected with other SARS-CoV-2 strains and were more likely to affect males. Meanwhile, the final clinical outcomes of children with Omicron-related FSs were generally favorable, but they seemed to have a more severe clinical course than those with traditional FSs or common type of Omicron infection. Thus, greater clinical vigilance for FSs in children (especially males) with Omicron infection is warranted. Furthermore, in children infected with the Omicron variant, FSs might be a clinical sign driven by direct viral invasion of the CNS in combination with inflammatory injuries mediated by the penetration of excessive proinflammatory mediators into the CNS. Finally, COVID-19 vaccination is likely to provide effective protection against FSs associated with Omicron infection in children. Intensification of vaccination coverage, particularly booster vaccination, among eligible children is needed.

## Data Availability

The original contributions presented in the study are included in the article/Supplementary Material, further inquiries can be directed to the corresponding author.
